# Genomic and expression analysis of multiple *Sry *loci from a single *Rattus norvegicus *Y chromosome

**DOI:** 10.1186/1471-2156-8-11

**Published:** 2007-04-04

**Authors:** Monte E Turner, Carey Martin, Almir S Martins, Jeffrey Dunmire, Joel Farkas, Daniel L Ely, Amy Milsted

**Affiliations:** 1 Department of Biology, The University of Akron, Akron, OH 44325-3908 USA; 2 Cuyahoga County Coroner's Office, Cleveland, OH 44106 USA; 3 Department of Physiology and Biophysics, Universidade Federal de Minas Gerais, ICB, Belo Horizonte, MG 31270-901 Brazil

## Abstract

**Background:**

*Sry *is a gene known to be essential for testis determination but is also transcribed in adult male tissues. The laboratory rat, *Rattus norvegicus*, has multiple Y chromosome copies of *Sry *while most mammals have only a single copy. DNA sequence comparisons with other rodents with multiple *Sry *copies are inconsistent in divergence patterns and functionality of the multiple copies. To address hypotheses of divergence, gene conversion and functional constraints, we sequenced *Sry *loci from a single *R. norvegicus *Y chromosome from the Spontaneously Hypertensive Rat strain (SHR) and analyzed DNA sequences for homology among copies. Next, to determine whether all copies of *Sry *are expressed, we developed a modification of the fluorescent marked capillary electrophoresis method to generate three different sized amplification products to identify *Sry *copies. We applied this fragment analysis method to both genomic DNA and cDNA prepared from mRNA from testis and adrenal gland of adult male rats.

**Results:**

Y chromosome fragments were amplified and sequenced using primers that included the entire *Sry *coding region and flanking sequences. The analysis of these sequences identified six *Sry *loci on the Y chromosome. These are paralogous copies consistent with a single phylogeny and the divergence between any two copies is less than 2%. All copies have a conserved reading frame and amino acid sequence consistent with function. Fragment analysis of genomic DNA showed close approximations of experimental with predicted values, validating the use of this method to identify proportions of each copy. Using the fragment analysis procedure with cDNA samples showed the *Sry *copies expressed were significantly different from the genomic distribution (testis p < 0.001, adrenal gland p < 0.001), and the testis and adrenal copy distribution in the transcripts were also significantly different from each other (p < 0.001). Total *Sry *transcript expression, analyzed by real-time PCR, showed significantly higher levels of *Sry *in testis than adrenal gland (p, 0.001).

**Conclusion:**

The SHR Y chromosome contains at least 6 full length copies of the *Sry *gene. These copies have a conserved coding region and conserved amino acid sequence. The pattern of divergence is not consistent with gene conversion as the mechanism for this conservation. Expression studies show multiple copies expressed in the adult male testis and adrenal glands, with tissue specific differences in expression patterns. Both the DNA sequence analysis and RNA transcript expression analysis are consistent with more than one copy having function and selection preventing divergence although we have no functional evidence.

## Background

The recent analysis of the DNA sequence of the human Y chromosome has required a change from the classical view of the mammalian Y chromosome as a passive, genetic graveyard to one of an active, self correcting entity [1,2]. In reality, the mammalian Y chromosome is a combination of both the classical and modern views, since different regions of the Y chromosome behave differently. Genes in the male specific region of the Y chromosome have been divided into three classes; X transposed, X degenerate, and ampliconic [3]. The palindromic structure of the ampliconic regions allow gene conversion to repair mutations in duplicate copies of the same locus much the same way as recombination for an autosomal chromosome [2], reducing divergence between copies. The X transposed and X degenerate regions act in the classical view of a non-recombining chromosome, accumulating mutations while either degrading or being maintained by function and selection.

One of the X degenerate loci on the mammalian Y chromosome is *Sry*. The *Sry *locus is conserved on mammalian Y chromosomes and is the transcription factor responsible for testis determination [4]. In comparisons of *Sry *amino acid sequences between mouse, human and other mammals the High Mobility Group (HMG) box region, responsible for DNA binding, is highly conserved while other regions show little if any conservation [5,6]. In humans, mutations in the HMG box result in sex reversal (XY females), while most mutations outside this region have normal sex-determination [7]. *Sry *may have functions other than testis determination, since *Sry *expression has been demonstrated from multiple adult male tissues and expression is conserved in human, mouse and rat [8-10]. Recent reports have demonstrated *Sry *activity in RNA splicing [11], in transcriptional control of the tyrosine hydroxylase promoter [12], blood pressure [13] and in brain function [10]. None of these reported activities is thought to be involved in testis determination.

While most mammals have only a single copy of *Sry*, multiple copies of the *Sry *locus on the Y chromosome have been found in some rodent species [14]. Whether these multiple copies are transcribed, translated and functional is not known. Since the X degenerate loci do not recombine or have the high levels of gene conversion observed for the ampliconic loci, mutations occurring in these *Sry *paralogs should become fixed and accumulate over time [15], leading to loss of function and decay. Thus the expectation for species with multiple copies of *Sry *on the Y chromosome would be for selection to maintain one copy for testis determination and any other copies would accumulate mutations and diverge or decay. The *Sry *HMG box region has been sequenced in 9 species of the family Microtidae (voles) and 8 of these species had multiple Y chromosome copies of *Sry*. Consistent with a decay hypothesis, some of the copies were obviously non-functional due to early, in frame stop codons or deletions causing frame-shift mutations [16]. In a comparison of partial *Sry *DNA sequences from six African murine species with multiple copies of the Sry locus, the pattern of variation and conservation of reading frame was consistent with these copies being functional [17]. The maintenance of multiple conserved copies could be the result of either functional constraints or recent duplication events creating copies that have not yet accumulated mutations and diverged. In the murine data multiple *Sry *orthologs were found in multiple species, thus these murine duplications are not recent. With the recent human Y chromosome data, another hypothesis for the conservation observed in the murine *Sry *copies is that these *Sry *loci are now organized and evolving like the ampliconic region of the Y chromosome, and the conservation and maintenance of reading frame are the result of gene conversion.

Previously, it was estimated from Southern blot densities that the laboratory rat, *Rattus norvegicus*, contained 4–5 copies of the *Sry *locus on the Y chromosome [14]. By sequencing paralogous copies from single Y chromosomes, hypotheses of gene conversion and/or functional constraints can be evaluated. Since the rat is an excellent model for physiological and functional studies, a characterization of *R. norvegicus Sry *loci can subsequently be used to evaluate both the expression and functional aspects of multiple copies of the *Sry *locus. In the present study we identify 6 full length *Sry *loci from a single *R. norvegicus *Y chromosome. The pattern of divergence and linkage disequilibrium within each copy is not consistent with gene conversion. Using differences between the copies we are able to determine which copies are expressed. The expression pattern is tissue specific and not all copies are expressed equally.

## Results

### Number of copies

Two primers were developed (3L and 02GR) from partial rat sequences in GenBank and preliminary rat *Sry *sequences from our lab. The amplification fragment includes the entire *Sry *coding region (510 bp) plus 5' (245 bp) and 3' (194 bp) flanking sequences. These primers amplify the correct size band from male genomic DNA and do no not amplify from female genomic DNA (data not shown). These primers were used to amplify genomic DNA from a single SHR/Akr male and the resulting amplification products cloned then sequenced. The sequence analysis of the 3L-02GR fragments identified 6 different copies of *Sry *from a single SHR/y male; *Sry1 *(GenBank:AY157669), *Sry2 *(GenBank:AY157670), *Sry3 *(GenBank AY157672), *Sry3B *(GenBank:AY157996), *Sry3B1 *(GenBank:AY157997) and *Sry3C *(GenBank:AY157671). The copy designations are based on insertion/deletion and/or repeat number differences, in addition to single unique base pair mutations (Table 1). The clones allowed comparison of 946 bp in *Sry1 *and *Sry3c*, 907 bp in *Sry2*, 942 bp in *Sry3 *and 948 bp in *Sry3B *and *Sry3BI*. Table 1 shows the diagnostic regions for identifying the six *Sry *copies.

**Table 1:** Sry Copy Diagnostic Regions

*Sry *copy	bp -71	bp 442–480	bp 466	bp 474	bp 550
Sry 1	C	+	G	(GCA)_8_	(GACC)_2_
Sry 2	C	Deletion	na	na	(GACC)_2_
Sry 3	C	+	G	(GCA)_8_	(GACC)_1_
Sry 3B	C	+	G	(GCA)_9_	(GACC)_1_
Sry 3B1	C	+	A	(GCA)_9_	(GACC)_1_
Sry 3C	TTCCT	+	G	(GCA)_8_	(GACC)_1_

Diagnostic regions used for the identification of the 6 different copies of *Sry *found in SHR/Akr. Base pair numbering is in relation to the sequence of *Sry1*. The GCA repeat at bp 474 and bp466 are part of the deletion present in *Sry2*.

### Comparison of copies

**Figure 1:**
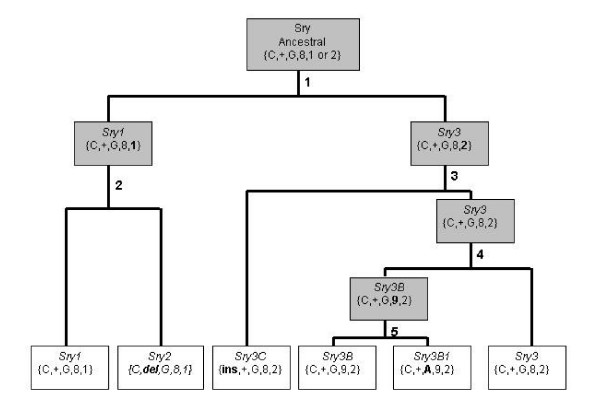
**Phylogeny of Sry Copies**. Phylogeny of the six *Sry *copies found on the SHR/Akr Y chromosome. Brackets {} indicate the genotype at the 5 diagnostic differences indicated in Table 1: bp-71, bp442–480, bp466, bp474 and bp 550 (del = deletion, ins = insertion, + = no deletion or insertion), bolded differences are new divergent mutations. Copies in shaded boxes are ancestral/hypothetical copies.

Table 2 summarizes the sequence differences between the copies; any insertion/deletion differences are counted as single differences. All these comparisons vary from between 907 to 948 bp depending on the copies being compared. The four "Sry3-like" copies (*Sry3*, *Sry3B*, *Sry3B1*, and *Sry3C*) differ from each other by only 2–7 differences (Table 2). These "Sry3-like" copies are more different from either *Sry1 *or *Sry 2 *(11–21 differences) than from each other. Values below the diagonal in Table 2 are the number of differences that occur in the 5' flanking region (26% of bases), coding region (54% of bases) or 3' flanking region (20% of bases). These differences are consistent with a single phylogeny of the relationship of the copies (Figure 1). Of the 28 differences between the copies only one is shared in a manner not consistent with the phylogeny.

**Table 2:** Differences between the Copies

Locus	*Sry1*	*Sry2*	*Sry3*	*Sry3B*	*Sry3B1*	*Sry3C*
*Sry1*		17	11	12	14	12
*Sry2*	5/9/3		18	19	18	21
*Sry3*	4/3/4	5/10/3		3	3	5
*Sry3B*	3/4/5	6/11/2	1/1/1		2	6
*Sry3B1*	4/5/5	5/11/2	0/2/1	1/1/0		8
*Sry3C*	5/3/4	8/10/3	5/0/0	4/1/1	5/2/1	

Differences between the six identified copies of *Sry *on the rat SHR Y chromosome for the 3L-02GR amplification fragment are above the diagonal. The number of the total differences in the a) 5' flanking sequence, b) coding region and c) 3' flanking region are listed below the diagonal (a/b/c). Insertion/deletion differences are counted as a single difference. Total bp in each comparison is different because of insertion/deletion differences changing the total base pairs compared in each. This table includes the diagnostic mutations in Table 1.

There are 28 differences (single nucleotide, insertion/deletion and repeat) identified between the copies and 18 of these are unique to a single copy. In the *Sry3 *duplicated copies, *Sry3 *and *Sry3B *have no unique differences consistent with having been duplicated (*Sry3 *twice and *Sry3B *once) (Fig. 1), while the duplicated copies have accumulated unique mutations as they have diverged from the original copy; *Sry3B1 *has 1 unique mutation and *Sry3C *has 3 unique mutations. Most of the unique differences are in *Sry1 *(6) and *Sry2 *(8) as they have diverged without duplication thereby accumulating unique mutations.

The open reading frame encodes a 169 amino acid protein in *Sry1, Sry3 *and *Sry3C*, a 170 amino acid protein in *Sry3B *and *Sry3B1*, and a 156 amino acid protein in *Sry2*. Table 3 shows the predicted amino acid sequence for each of the 6 Sry copies and compares them to the amino acid sequence of the HMG box region for both human and mouse. Since conservation between species outside of the HMG box is reduced, only the HMG region is included in the *M. musculus *or human *SRY *comparisons in Table 3.

**Table 3:** Predicted Amino Acid Sequence Comparison

Sry1	MEGHVKRPMN AFMVWSRGER RKLAQQNPSM QNSEISK**H**LG YQWKSLTEAE
Sry2	**Q** M GE **H** L QQ S Q S Q YQ S
Sry3	H M GE R L QQ S Q S Q YQ S
Sry3B1	H M GE R L QQ S Q S Q YQ S
Sry3b	H M GE R L QQ S Q S Q YQ S
Sry3C	H M GE R L QQ S Q S Q YQ S
*M. musculus*	H M GE **H** L QQ S Q **T** Q **CR** S
Human	H **I** **DQ** R **M** **LE** **R** **R** S Q YQ **M**
	
Sry1	KRPFFQEAQR LKTLHREKYP NYKYQ**P**HRRV KVPQRSYTLQ REVASTKLYN
Sry2	R R KTL **P**
Sry3	R R KTL T
Sry3B1	R R KTL T
Sry3B	R R KTL T
Sry3C	R R KTL T
M.m	R R K**I**L
Human	**W** **K ** **QAM**
	
Sry1	LLQWDNNLHT IIYGQDWARA AHQSSKNQKS IYLQPVDIPT GYPLQQKQQH
Sry2	**(--**
Sry3	
Sry3B1	
Sry3B	
Sry3C	
	
Sry1	QQQQHVHLQQ QQQQQHQFH
Sry2	**----------)**
Sry3	H
Sry3B1	**M** **[Q]**
Sry3B	H **[Q]**
Sry3C	H

Predicted amino acid sequence for each of the 6 copies of *Sry *found on the SHR Y chromosome compared to the amino acid sequence of *Sry1*. The HMG box (amino acids 5–73) is underlined and compared to *Mus musculus *and Human. Human (nm_003140.1) and mouse (nm_11564.1) sequences are from REFSEQ. Only differences are noted, (---) indicate amino acids that are deleted in *Sry2*, and [] indicate amino acids added in *Sry3B *and *Sry3B1*. Consensus rat sequence is in black and differences are **bold**.

**Figure 2:**
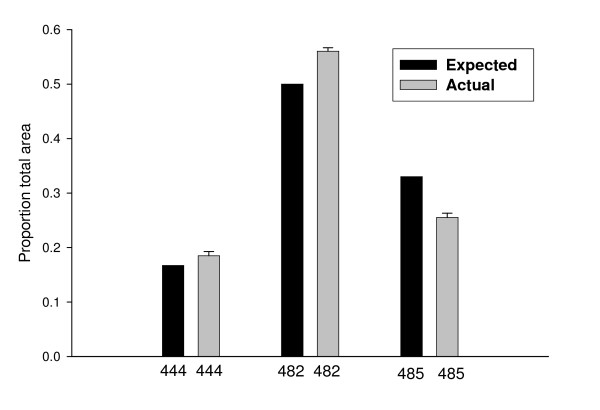
**Fragment Analysis of SHR Genomic DNA**. Proportion of total area of three peaks (444, 482, 485) in each individual peak. The expected results are calculated assuming 6 copies of the Sry locus as identified in this paper. The actual values are averaged from two replicates from each of 5 different males, means plus SD. 444 = 444 bp peak resulting from Sry2, 482 = 482 bp peak resulting from Sry1, Sry3 and Sry3C, and 485 = 485 bp peak resulting from Sry3B and Sry3BI.

**Figure 3:**
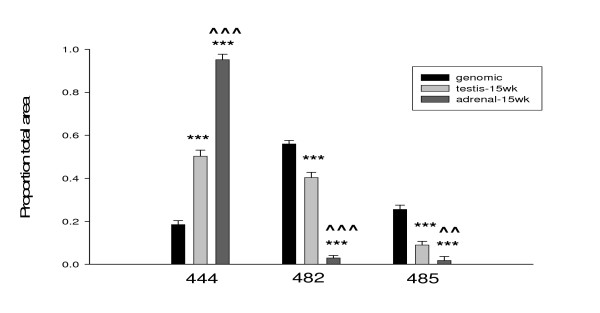
**Fragment analysis of cDNA samples**. Comparison of peak proportions from analysis of 15 week old testis and adrenal cDNA samples. Proportion of total area of three peaks (444, 482, 485) in each individual peak is compared to those values from genomic DNA. 444 = 444 bp peak resulting from *Sry2*, 482 = 482 bp peak resulting from *Sry1*, *Sry3 *and *Sry3C*, and 485 = 485 bp peak resulting from *Sry3B *and *Sry3BI*. Each peak is the average of 2 replications from 5 individuals, means, +/- s.e.m.,*** = p < .001 compared to genomic, ^^ = p < . 01, ^^^ = p < .001 adrenal vs. testis (t-tests).

After amplification of genomic DNA using fluorescently labeled primers (NED-P1mod and S1502GLrev), the 6 copies have three different length amplicons; 444 bp (*Sry2*), 482 bp (*Sry1*, *Sry3*, *Sry3C*) and 485 (*Sry3B *and *Sry3BI*). When genomic DNA from the original SHR/y strain is used for amplification and the products separated by capillary electrophoresis the three expected peaks are seen and there are no peaks present in female samples (data not shown). Figure 2 indicates the average area of each of the three size peaks averaged from 5 different SHR/y males and the expected proportion for each peak assuming 6 copies of *Sry*. By comparing the areas of the three peaks with the number of predicted copies in that peak we see a close approximation to the expected values (Figure 3). Any differences between the observed and expected areas may be the result of inherent amplification differences between the copies but also the possibility there may be other as yet unidentified copies. For instance an extra unidentified *Sry1 *copy would increase the number of loci contributing to the 482 bp peak from three to four and change the expectation from 0.50 (3/6) to 0.57 (4/7).

### Transcript Analysis

Next, we used the fragment analysis procedure on cDNA prepared from mRNA transcripts rather than genomic DNA to estimate the proportion of the copies present in an RNA sample. This does not quantify the absolute amount of *Sry *RNA in the sample but it allows us to partition the total *Sry *mRNA into copies. Figure 3 shows the proportion of each peak from testis and adrenal gland, averaged from 5 males 15 weeks of age. The proportion of the peaks from testis and adrenal cDNA are both significantly different from the genomic distribution for each *Sry *type (444,482,485, testis p < .001, adrenal p < .001) and the testis and adrenal peak levels are significantly different from each other for each *Sry *type (444,482,485, p < .01–.001).

**Figure 4:**
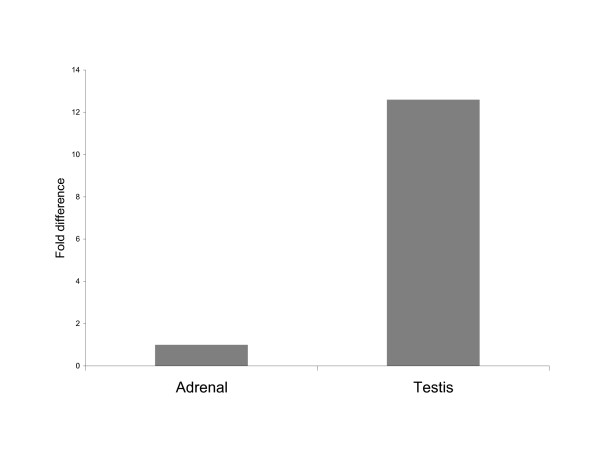
**Real-time RTPCR from testis and adrenal**. Relative proportions of *Sry *mRNA in the testis and adrenal glands of SHR/y males (n = 5 per group). S26 ribosomal RNA was used as control transcript to normalize *Sry *levels. Mean ΔC_T _*Sry *(adrenal gland) = 11.54 +/- 0.30 and mean ΔC_T _*Sry *(testis) = 11.54 +/- 0.30. Fold difference, Sry (testis) compared Sry (adrenal gland) = 2^-(7.88–11.54) ^= 12.6X.

Real-time RT-PCR for 15 week male rat testis and adrenal are presented in Figure 4. Total *Sry *levels in the testis were significantly higher (12.6X, p, 0.001) the levels in the adrenal.

## Discussion

The identification of six copies of the *Sry *locus on the rat SHR/y Y chromosome is consistent with the prediction of 4–5 copies by Nagamine [14]. Nagamine used Southern blot autoradiographs to estimate copy number, while we have used DNA sequence differences to identify the copies. The close agreement between our results and Nagamine's, using different methods, would argue that there are not additional unidentified copies or additional duplicate copies with identical sequence. Previous rat *Sry *sequences in GenBank have only been identified as *Sry *without regard to the existence of multiple copies. Using the diagnostic regions identified for the 6 copies (Table 1), we can determine the identity of each pre-existing rat Sry sequence in GenBank; GenBank X89730*Sry2*, GenBank Z26907*Sry2*, GenBank AJ222688*Sry3C*, GenBank CR759961*Sry3C*. Because we used primers spanning the coding region to amplify and clone these sequences, it is possible that other copies with either a partial coding region or mutations that affect primer binding exist but were not identified.

In a comparison of partial *Sry *DNA sequences in 6 murine species with multiple copies of *Sry *(2–4 copies/species), sequence divergence between paralogous copies in different species varied from 1% to 8% [17]. All copies had a long open reading frame with a stop codon, similar to *M. musculus *which has one copy of *Sry*. Lundrigan and Tucker [17] interpreted this conservation of reading frame and HMG box to indicate that these paralogous copies are functional. While other comparisons of *Sry *have used only partial Sry sequences, we have compared the entire coding region along with regions from both 5' and 3' flanking sequences. Our results are similar to the murine results as the six copies differ by 0.2 to 2% and have a conserved reading frame, but this is within a single Y chromosome and not between Y chromosomes in different species. The rat Y chromosome analysis is unique in finding 6 divergent copies on a single Y chromosome These copies from the same Y chromosome differ from each other by as much as some *Sry *loci in different species of *Mus *(with a single copy of *Sry*) [5,6]. Thus, some of these duplications cannot be of recent origin.

For six different copies to be present and identified, two separate processes must have occurred on the rat Y chromosome. First an ancestral copy must have duplicated and then copies diverge through the accumulation of mutations. We can only identify the duplicate copies by their divergent mutations. Figure 1 demonstrates a phylogeny consistent with the sequence data from the 6 copies. This phylogeny is based on the insertion/deletion/repeat differences and single base pair differences between the copies. For example, all of the *Sry3 *related copies (*Sry3*, *Sry3B*, *Sry3B1*, and *Sry3C*) have one copy of the GACC repeat at bp 550 while *Sry1 *and *Sry2 *have two copies (Table 1). The phylogeny requires 5 duplications from a single ancestral copy. The divergences show *Sry1*, *Sry2 *and *Sry3 *to be the oldest copies. The Sry3-like loci are the result of three duplications while *Sry1 *and *Sry2 *have not been duplicated. This could indicate that the Sry3-like loci are located in an ampliconic region of the Y chromosome where duplications are more probable.

Is there evidence for gene conversion in the *Sry *copies? Gene conversion would be identified by a reduction in genetic divergence between copies but also by a decay of linkage disequilibrium within the mutations on a single copy. In these comparisons we are referring to the linkage disequilibrium of the specific mutations within a single copy rather than linkage disequilibrium between copies, which would require information about the location of each copy on the Y chromosome. Gene conversion would decay linkage disequilibrium between differences in the same copy and shared mutations between copies would be based on gene conversion events rather than the phylogeny. Because of the duplication history of the copies, there are three comparisons that are instructive: between the four Sry3-like copies, between *Sry1 *and *Sry2*, and between *Sry1*/*Sry2 *and the Sry3-like copies. The four Sry3-like copies have high genetic identity but the only difference shared between all the Sry3-like copies is the GACC repeat number (Table 1), the other 8 differences between these copies are unique to a single copy. There has been no decay of linkage disequilibrium within these copies, so although our precision to identify gene conversion is small, there is no indication of gene conversion in the Sry3-like copies. The accumulation of unique differences in *Sry1 *and *Sry2 *would not be consistent with gene conversion. Between the Sry3-like copies and *Sry1*/*Sry2 *there is only one difference shared between copies that is not consistent with the phylogeny, a single base difference shared between *Sry1*, *Sry3C *and *Sry3B *(bp -154). Other differences near this base pair are not shared. Although we cannot say gene conversion has not occurred, the patterns are not consistent with gene conversion being a major force in preventing genetic divergence between these copies.

One of the diagnostic regions between the copies (Table 1) is the different number of GCA trinucleotide repeats in the *Sry *coding region (bp 474). These trinucleotide repeats occur as a result of polymerase slippage and have a high mutation rate (10^-3^) on the human Y chromosome [18]. Are these repeat differences unique events in this lineage? If a repeat expansion/contraction to the same length occurred more than once in the same lineage then flanking markers would not be in linkage disequilibrium with repeat length and would show a pattern that could be interpreted as gene conversion. The data are consistent with the two repeat differences between copies being unique events in this phylogeny (Table 1).

Table 3 shows the predicted amino acid sequence for each of the 6 rat *Sry *copies and compares the conserved HMG box region for human and mouse. Of the predicted *Sry *amino acid sequence differences among the copies seen in the HMG box region of the protein (amino acids 5–73) (Table 3), only one difference (amino acid 38) in *Sry1 *could potentially affect function. *Sry1 *has a histidine residue at amino acid position 38 while all other identified rat Sry copies, as well as *M. musculus *Sry and human *SRY*, have a glutamine residue (Table 3). Although this is a conserved amino acid change (both are polar), it could affect DNA binding. All other HMG box region amino acid differences identified in the six rat *Sry *loci are also found in either the human or mouse HMG amino acid sequence and therefore we assume them to be consistent with DNA binding and function.

None of the insertion/deletion differences between the copies disrupts the carboxy terminal portion of the *Sry *protein, as all are in frame. In comparisons of the DNA sequence between the six rat copies, the *Sry2 *locus has the most unique base pair differences (8), and although seven of these occur in the coding region, only two result in amino acid changes. One of the two amino acid differences found in *Sry2 *is consistent with the amino acid found in *Mus musculus *and could be the ancestral condition rather than a diverged mutation.

There are no published data on expression for any *Rattus Sry *copy in GenBank or expression database. An anomaly of mammalian *Sry *expression is the absence of *Sry *sequences in all EST expression databases, although EST libraries from tissues such as the testis, where *Sry *expression has been demonstrated by northern and western blots, have had significant number of clones sequenced. The *Sry *transcript may have a secondary structure which prevents or reduces its cloning potential from mRNA samples. GenBank X89730 is identified in GenBank as a cDNA sequence, but the original publication [19] indicates that the "cDNA" designation was the authors designation for DNA amplified with a Taq polymerase from genomic DNA, not from cDNA. All of our sequence prior to the coding region is probably 5' UTR, given the identified transcriptional start sites in mouse and human *Sry *[8,20]. The only copy with significant variation in the sequenced 5' flanking region is *Sry3C*, which includes the diagnostic insertion at bp -71. The relationship between *Sry3C *and the other *Sry3 *copies is skewed toward differences in the 5' flanking region (Table 2) and could potentially alter the expression profile or stability of this copy. Over 70% of the differences in *Sry3C *are in the 5' flanking region, which represents only 25% of the total sequence compared (249/982).

To examine expression of the *Sry *copies, we developed a modification of the fluorescent marked capillary electrophoresis method normally used to genotype microsatellites or identify loss of heterozygosity in tumor samples. Two primers spanning the deletion in *Sry2 *and the repeat differences in the coding region give three different sized amplification fragments; 444 bp (*Sry2*), 482 bp (*Sry1*, *Sry3*, and *Sry3C*), 485 bp (*Sry3B *and *Sry3BI*). The total area of the three peaks and the proportion in each peak can be used to estimate the relative proportion of each grouping in a DNA or cDNA sample. Figure 2 shows the average proportion of each peak from genomic DNA of 5 different SHR/y males. The expected values for each of the peaks are determined from how many of the 6 copies are present in that peak and only one copy of each is present. The proportions in each peak are consistent between males and close to the expected values. Slight differences in amplification efficiency may account for the consistent underestimate of the 485 peak. The primers used for the fragment analysis were different from, and located in regions different from, the primers used to amplify and clone the *Sry *sequences used in the DNA sequences. The close agreement between the fragment analysis and the prediction of 6 copies from genomic DNA would argue that the SHR Y chromosome may not contain other full length *Sry *copies.

This fragment analysis procedure was then used to identify copies from cDNA samples prepared from RNA isolated from SHR/y adult male testis and adrenal glands (Figure 3). *Sry *expression in adult testis has been identified in human and *Mus *adult males, both of which have only a single copy of Sry [8,9]. We earlier demonstrated that *Sry *increases the transcriptional activity of the tyrosine hydroxylase promoter in vitro [12] and that *Sry *and *Th *mRNA are both present in the rat adrenal medulla [12] consistent with a functional relationship between *Sry *and tyrosine hydroxylase (the rate limiting enzyme for catecholamine synthesis). The expression results from the cDNA samples are different from the genomic samples; the 444 bp peak (*Sry2*) is the major transcript in the testis, with less of the 482 bp peak (*Sry1*, *Sry3*, and *Sry3C*) and 485 bp peak (*Sry3B*, and *Sry3BI*) (Figure 3). The adrenal transcript proportions are very different from the testis proportions: they are almost completely 444 bp peak (*Sry2*) (Figure 3). These data demonstrate that more than one copy is expressed and that there are tissue specific expression patterns. The fragment analysis provides the proportion of each of the copy groups relative to the total *Sry *expression; it does not show the total amount of *Sry *expressed. Analysis of total *Sry *transcript levels by real-time RT-PCR (Figure 4) showed about 13 times more *Sry *transcripts in the testes than in adrenal gland. It is unlikely that the *Sry *genes are expressed at such levels (total *Sry *transcripts in testis represent about 0.4% of the levels of the invariant ribosomal S26 transcript) unless they are playing a role in some physiological process. The difference in total *Sry *expression in testis compared to adrenal gland, in combination with the differences seen in relative proportions of individual transcripts after fragment analysis, is consistent with different *Sry *products impacting more than one phenotype in adult male rats.

Although historically, the only function for *Sry *was testis determination both the DNA sequence analysis and RNA transcript expression analysis are consistent with more than one copy being functional and selection preventing divergence. We do not have evidence of additional functions from these results but there have been recent publications that extend the view of potential Sry interactions [10-12]. It is evident from sex reversed individuals with *Sry *mutations that any functions of *Sry *in addition to testis determination are not essential or do not have large phenotypic consequences. Does this mean that the presence of *Sry *transcripts and protein have no effect in adult male tissues? Electroporation of a *Sry1 *expression vector into the rat adrenal gland increased blood pressure 21 days after the electroporation [13]. This result demonstrates that *Sry *expression in a tissue can have physiological or phenotypic consequences. Whether this result is a byproduct of an inherent function or a modification because *Sry *is a transcription factor that may affect other pathways when it is present in a tissue cannot be determined at this point. Could any such small phenotypic modifications be maintained by selection? As a single autosomal locus a small phenotypic effect may not reach a threshold high enough to be controlled by anything other than stochastic processes. But with an entire Y chromosome inherited as a unit, a group of small, each alone, inconsequential but together add enough to be selected. The classic evolutionary studies with natural populations and inversions in *Drosophila pseudoobscura *demonstrate the potential selective consequences of grouping genes together [21]. These results are consistent with selection, and the evidence of *Sry *effects other than testis determination, opens the possibility that *Sry *can have phenotypic effects in addition to testis determination. This hypothesis can be evaluated in systems such as *Rattus norvegicus *and then tested in other mammals.

## Conclusion

The SHR Y chromosome contains at least 6 full length copies of the *Sry *gene. These copies have a conserved coding region and conserved amino acid sequence. The pattern of divergence is not consistent with gene conversion as the mechanism for this conservation. Expression studies show multiple copies expressed in the adult male testis and adrenal glands, with tissue specific differences in expression patterns.

## Methods

### Rat Strains

Male SHR/y:Akr (Spontaneously Hypertensive Rat Y chromosome congenic strain) rats from the University of Akron breeding colonies. All animal protocols were approved by the Institutional Animal Care and Use Committee of the University of Akron (IACUC:proposal # 03-03A).

### PCR reactions and cloning

The 3L (5' AATCCACAGGGGTTTTGGTT) and 02GR (5'GGGAGCAGGCCCTTTATTAC) primers were used to amplify genomic DNA from a single male (temperature cycling: 94° 4 minutes; 30 cycles of 94° 1 minute, 60° 1 minute, 72° 1 minute; 72° 7 minutes). Amplification was accomplished using *TaqPlus*^® ^Long polymerase, a mix of *Taq *and *Pfu *DNA polymerases, a mixture with proofreading capabililty (Stratagene, Carlsbad, CA). The resulting PCR products were cloned into the pCR^® ^4-TOPO^® ^(Invitrogen Corp., Carlsbad, CA) vector using the TOPO^® ^TA Cloning and Transformation kit (Invitrogen Corp., Carlsbad, CA) according to manufacturer's specifications. Two independent PCR amplifications were performed and each amplification was independently cloned and transformed, and then 40 clones from each amplification library were sequenced.

### DNA sequencing and sequence analysis

An ABI Prism 310 Genetic Analyzer (Applied Biosystems, Foster City, CA) and BigDye™ Terminator Cycle Sequencing chemistry were used to sequence all DNA samples. Sequencing reactions were prepared as listed in Applied Biosystem's Chemistry Guide for BigDye Terminators. Sequences were aligned and analyzed with Sequencher sequence analysis software (Gene Codes Corp. Inc., Ann Arbor, MI).

### RNA Isolation and transcript analysis

Total cellular RNA was isolated with RNA STAT-60 (TEL-TEST), treated with DNase to remove any contaminating genomic DNA, and used as template with avian reverse transcriptase (enhanced avian HS RT, Sigma) and a mix of random nonamer primers to generate cDNAs. RNase inhibitor (SUPERase•In, Ambion) was included to insure RNA integrity. A no-RT control was included for each sample.

### cDNA First Strand Synthesis and RT-PCR

500 ng of DNased RNA was used as template for M-MLV reverse transcriptase (ArrayScript, Ambion) and primed using the Sry specific oligonucleotide RTallS-A (5'-GGACAGTAAGTAGGTTAGCT-3'). Rnase inhibitor (SUPERase*IN, Ambion) was added to prevent RNA degradation during cDNA synthesis. Dnased RNA, water, and primer were initially heated to 70°C for 10 minutes for denaturation of transcript secondary structure. The addition of 10 mM dNTP, Rnase inhibitor, RT enzyme and buffer was followed by incubation at 25°C for 15 minutes and 44°C for 1 hour. The RT enzyme was heat inactivated at 95°C for 5 minutes and immediately placed on ice. For every sample that was reverse transcribed, a control no-RT tube was prepared. RT-PCR was carried out immediately following generation of first strand cDNA. The primer set RTallS-B (5'-AGTAGGTTAGCTGCTGCTAG-3') and 5'S2L (5'-CCATCTCTGACTTCCTGGTTG-3') was used to amplify all Sry copies from the cDNA template. Thermocycler conditions were as follows: 94°C for 4 min, 25 cycles of 94°C for 1 min, 59°C for 1 min, 72°C for 1 min, followed by 72°C for 10 min. In addition to the no-RT control, a no template negative control and genomic positive control were included.

### Real-time RT-PCR

For real-time RT-PCR, primers for Sry and S26 (an invariant transcript encoding ribosomal protein 26 in the small ribosomal subunit of rat) were selected using Primer Express software (Applied Biosystems). Primers (300 μM each), 50–100 ng of cDNA and SYBR Green PCR Master Mix (Applied Biosystems) were combined and run under standard conditions in an ABI Prism 7700 Sequence Detection. In all assays no-RT samples and no-template PCR controls were included. After primer efficiency was determined, for each sample the threshold cycle (C_T_) was determined. C_T _values for the S26 normalizer were subtracted from C_T _values for experimental samples to obtain ΔC_T _values. Transcript levels in the adrenal gland were expressed as fold difference relative to testis values (2^-ΔΔCT^). Male-specific real-time PCR primers for Sry (SryL, 5'-TGG GAT TCT GTT GAG CCA ACT-3' and SryR, 5'-GCG CCC CAT GAA TGC AT-3') detect all Sry transcripts.

### Generation of Fluorescent Labeled Fragments

Products of the RT-PCR reaction were used as template in subsequent PCR reactions each with a primer set containing one 5'fluorescent labeled and one unlabeled oligonucleotide. The primer set NED-P1mod (5'-NED-GAATGCATTTATGGTGTGGTCCCG-3') and S1502Glrev (5'-TAGTGGAAC TGGTGCTGCTG-3') was used to produce amplicons of three different lengths. Thermocycler conditions were as follows: 94°C for 4 min, 30 cycles of 94°C for 1 min, 61°C for 1 min, 72°C for 1 min, followed by 72°C final extension for 20 min. The fluorescent dye NED was incorporated at the 5' end of each amplicon and the size of the products were 444 bp, 482 bp, and 485 bp corresponding to Sry2, Sry1/3/3C, and Sry3B/3B1 respectively.

Because the *Sry *copies all contain a trinucleotide repeat region there is also a stutter band 3 bp smaller than the predicted sizes. Because the stutter for the 485 peak would run with the 482 peak overestimating the 482 area, we have made a simple correction using the area of the 479 peak (stutter from 482). The ratio of the 479 to 482 areas is multiplied by the 485 area and this result is subtracted from the 482 area (Corrected Area482 = Area482-({Area479/Area482} × Area485)).

### Preparation of Samples for Fragment Analysis

Fluorescent labeled PCR products were diluted in water 1:10. Each well of the final analysis plate contained 1 μl of diluted PCR product, 0.4 μl of sizing standard (GeneScan 600LIZ, Applied Biosystems), and 8.6 μl of formamide (Hi-Di Formamide, Applied Biosystems). The no-RT, no template control, and genomic samples were included as controls. Samples were processed using the ABI 3130 × 1 Genetic Analyzer (Applied Biosystems) and fragments were analyzed using GeneMapper v4.0 software (Applied Biosystems).

## Authors' contributions

MET, DLE and AM designed and directed studies, were involved in the analysis of the data, and writing of manuscript. ASM developed primers and PCR procedures. CM did the cloning and sequencing of the 3L-02GR amplification fragment. JD and JF did the fragment analysis from genomic DNA and cDNA. All authors have read and approved manuscript.

## References

[B1] Burgoyne PS (1998). The mammalian Y chromosome: a new perspective. BioEssays.

[B2] Rozen S, Skaletsky H, Marszalek JD, Minx PJ, Cordum HS, Waterston RH, Wilson RK, Page DC (2003). Abundant gene conversion between arms of massive palindromes in human and ape Y chromosomes. Nature.

[B3] Skaletsky H, Kuroda-Kawaguchi T, Minx PJ, Hillier HSL, Brown LG, Repping S, Pyntikova T, Ali J, Delahunty K, Du H, Fewell G, Fulton G, Graves T, Hou SF, Latrielle P, Leonard R, Maupin R, Miner T, Nash W, Nguyen C, Ozersky P, Pepin K, Rock S, Rohlfing T, Scott K, Stoneking C, Strong C, Tin-Wollam A, Waterston RH, Wilson RK, Rozen S, Page DC (2003). The male-specific region of the human Y chromosome: A mosaic of discrete sequence classes. Nature.

[B4] Koopman P, Gubay J, Vivian N, Goodfellow P, Lovell-Badge R (1991). Male development of chromosomally female mice transgenic for Sry. Nature.

[B5] Lundrigan BL, Tucker PK (1994). Tracing paternal ancestry in mice, using the Y-linked sex-determining locus, Sry. Mol Bio Evol.

[B6] Nachman MW, Aquadro CF (1994). Polymorphism and Divergence at the 5' flanking region of the sex-determining locus, Sry, in mice. Mol Biol Evol.

[B7] Schafer AJ, Goodfellow PN (1996). Sex determination in humans. BioEssay.

[B8] Clepet C, Schater AJ, Sinclair AH, Palmer MS, Lovell-Badge R, Goodfellow PN (1993). The human *SRY *transcript. Hum Mol Genet.

[B9] Rossi P, Dolci S, Albanesi C, Grimaldi P, Geremia R (1993). Direct evidence that the mouse sex-determining gene Sry is expressed in the somatic cells of male fetal gonads and in the germ cell line in the adult testis. Mol Reprod Dev.

[B10] Dewing P, Chiang CW, Sinchak K, Sim H, Fernagut PO, Kelly S, Chesselet MF, Micevych PE, Albrecht KH, Harley VR, Vilain E (2006). Direct regulation of adult brain function by the male-specific factor SRY. Curr Biol.

[B11] Ohe K, Lalli E, Sassone-Corsi P (2002). A direct role of SRY and SOX proteins in pre-mRNA splicing. Proc Natl Acad Sci USA.

[B12] Milsted A, Serova L, Sabban EL, Dunphy G, Turner ME, Ely DL (2004). Regulation of tyrosine hydroxylase gene transcription by Sry. Neurosci Lett.

[B13] Ely D, Milsted A, Bertram J, Ciotti M, Dunphy G, Turner ME Sry delivery to the adrenal medulla increases blood pressure and adrenal medullary tyrosine hydroxylase of normotensive WKY rats. BMC Cardiovasc Disord.

[B14] Nagamine CM (1994). The testis-determining gene, *SRY*, exists in multiple copies in Old World rodents. Genet Res Camb.

[B15] Steinemann M, Steinemann S, Lottspeich F (1993). How Y chromosomes become genetically inert. Proc Nat Acad Sci USA.

[B16] Bullejos M, Sanchez A, Burgos M, Jimenez R, Diaz de la Guardia R (1999). Multiple mono- and polymorphic Y-linked copies of the SRY HMG-box in Microtidae. Cytogenet Cell Genet.

[B17] Lundrigan BL, Tucker PK (1997). Evidence for multiple functional copies of the male sex-determining locus *Sry *in African murine rodents. J Mol Evol.

[B18] Kayser M, Kittler R, Erler A, Hedman M, Lee AC, Mohyuddin A, Mehdi SQ, Rosser Z, Stoneking M, Jobling MA, Sajantila A, Tyler-Smith C (2000). Characteristics and frequency of germline mutations at microsatellite loci from the human Y chromosome, as revealed by direct observation in Father/Son pairs. Am J Hum Genet.

[B19] An J, Beauchemin N, Albanese J, Abney TO, Sullivan AK (1997). Use of a rat cDNA probe specific for the Y chromosome to detect male-derived cells. J Androl.

[B20] Margarit E, Guillen A, Rebordosa C, Vidal-Taboada J, Sanchez M, Ballesta F, Oliva R (1998). Identification of Conserved Potentially Regulatory Sequences of the SRY Gene from 10 Different Species of Mammals. Biochem Biophys Res.

[B21] Anderson W, Arnold J, Baldwin D, Beckenbach A, Brown C, Bryant S, Coyne J, Harshman L, Heed W, Jeffery D, Klaczko L, Porter J, Powell J, Prout T, Schaeffer S, Stephens J, Taylor C, Turner M, Williams G, Moore J (1991). Four Decades of inversion polymorphism in *Drosophila pseudoobscura*. Proc Nat'l Acad Sci USA.

